# Magnesium: The Forgotten Electrolyte—A Review on Hypomagnesemia

**DOI:** 10.3390/medsci7040056

**Published:** 2019-04-04

**Authors:** Faheemuddin Ahmed, Abdul Mohammed

**Affiliations:** 1 OSF Saint Anthony Medical Center, 5666 E State St, Rockford, IL 61108, USA; 2 Advocate Illinois Masonic Medical Center, 833 W Wellington Ave, Chicago, IL 60657, USA; drmubeen@gmail.com

**Keywords:** magnesium, proton pump inhibitors, diuretics, hypomagnesemia

## Abstract

Magnesium is the fourth most abundant cation in the body and the second most abundant intracellular cation. It plays an important role in different organ systems at the cellular and enzymatic levels. Despite its importance, it still has not received the needed attention either in the medical literature or in clinical practice in comparison to other electrolytes like sodium, potassium, and calcium. Hypomagnesemia can lead to many clinical manifestations with some being life-threatening. The reported incidence is less likely than expected in the general population. We present a comprehensive review of different aspects of magnesium physiology and hypomagnesemia which can help clinicians in understanding, identifying, and treating this disorder.

## 1. Introduction

Magnesium is one of the most abundant cation in the body as well as an abundant intracellular cation. It plays an important role in molecular, biochemical, physiological, and pharmacological functions in the body. The importance of magnesium is well known, but still it is the forgotten electrolyte. The reason for it not getting the needed attention is because of rare symptomatology until levels are really low and also because of a lack of proper understanding of magnesium physiology. Some studies estimate that approximately three-fourths of Americans do not take the recommended dietary allowance of magnesium [1]. The reported incidence of hypomagnesemia is likely less than expected. The reported incidence of hypomagnesemia is approximately 2% in the general population. In hospitalized patients, the risk is the highest for intensive care unit (ICU) patients [2]. The cause of hypomagnesemia depends primarily on alterations in intake, redistribution, and excretion. Understanding the physiological aspects is important to guiding the management of magnesium disorders.

## 2. Physiology

### 2.1. Absorption

Based on literature review, magnesium is absorbed mostly in the small intestine and to some extent in the large intestine [3]. There are two pathways, namely, paracellular and transcellular, for magnesium absorption. Paracellular absorption is the passive transport which is responsible for 80–90% of uptake [4].

The exact mechanism for paracellular transport is not known but has been attributed to high luminal magnesium concentration and tight junction permeability regulated by claudins. Active transport is facilitated by transient receptor potential channel melastatin member 6 (TRPM6) and TRPM7 Mg^2+^ channels [5]. Although the mechanism of regulatory factors driving magnesium absorption is not clear, intestinal absorption is not directly proportional to dietary intake but is also dependent on magnesium status in the body.

A lower dietary magnesium intake will lead to reliance on active transcellular uptake via magnesium-specific transporters in the large intestine, namely, TRPM6 and TRPM7 [6]. Hypomagnesemia in patients with familial hypomagnesemia with secondary hypocalcemia has been attributed to mutations of TRPM6 [7].

### 2.2. Storage and Distribution

Around 99% of the magnesium in the body is in storage form and less than 1% is in serum and red blood cells [8]. Bone tissue is the largest store of magnesium in the body. Approximately one-third of this is concentrated on the bone surface and is related to the serum magnesium concentration [4]. The remaining magnesium is found in skeletal muscles and soft tissues. Since around 0.3% of the total body magnesium is in serum, serum magnesium estimations may not accurately reflect the status of magnesium stores [8]. Serum magnesium is present in three states: two-thirds in ionized form, one-third is protein bound mostly to albumin, and a very small state complexed to anions.

### 2.3. Excretion/Elimination

Kidneys play a major role in magnesium homeostasis. Under normal physiological conditions, around 90–95% of the filtered magnesium is reabsorbed with only 3–5% excreted in the urine. Approximately 15–20% of the filtered magnesium is absorbed in the proximal convoluted tubule. The thick ascending limb (TAL) of the loop of Henle is the major site for reabsorption with 65–75% being reabsorbed here. A small percentage is reabsorbed in the distal small tubule [9]. 

#### 2.3.1. Thick Ascending Loop

The process of reabsorption of magnesium in the thick ascending loop is facilitated by paracellin-1/claudin 16 and claudin 19 belonging to the claudin protein family. There are two conditions required for magnesium reabsorption in TAL: first is a lumen positive transepithelial voltage and second is paracellular permeability for the divalent cations. In TAL, sodium, potassium, and chloride enter the cell through Na-K-2Cl contransporter (NKCC2) in the luminal membrane. Sodium then exits the cell through Na/K ATPase present at the basolateral membrane. In this exchange, potassium that has entered the cell is secreted into the lumen through the renal outer medullary potassium channel. Chloride leaves the cell through the basolateral membrane via chloride channels. With continuous NaCl reabsorption along TAL, a lumen positive voltage gradient is created. This is required for the paracellular permeability of divalent cations like magnesium and calcium [10].

Loop diuretics by inhibiting NKCC2 therefore result in increased magnesium excretion. Familial hypercalciuric hypomagnesemia with nephrocalcinosis (FHHNC) is an autosomal recessive disorder with mutations in the genes encoding claudin 16 and 19. This disorder is associated with excessive magnesium and calcium excretion resulting in hypomagnesemia, hypercalciuria, and nephrocalcinosis [10]. Barrter’s syndrome is a genetic disorder characterized by defective NaCl reabsorption in the TAL. There are six different types of this syndrome based on the gene affected. It presents as hypokalemia, hypochloremia, and metabolic alkalosis [11].

#### 2.3.2. Distal Convoluted Tubule

**Figure 1:**
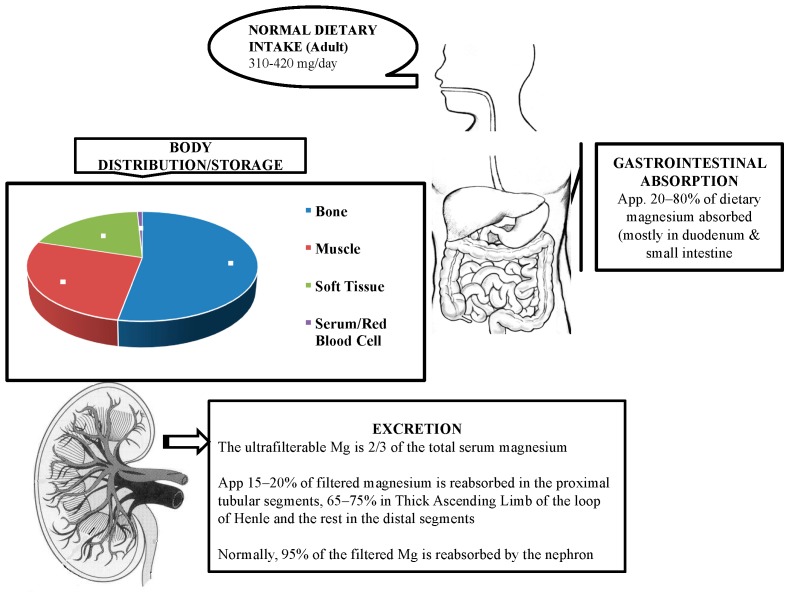
Physiology of magnesium.

Five to ten percent of filtered magnesium is reabsorbed from the distal convoluted tubule (DCT) segment. The mode of absorption here is through an active transport via the apical TRPM6 pathway. Amiloride acts at the DCT and is not only potassium-sparing but also a magnesium-sparing diuretic. The exact mechanism by which amiloride decreases magnesium excretion is unclear. Gitelman syndrome is an autosomal recessive disorder caused by mutation in the gene that encodes thiazide-sensitive NaCl cotransporter in the DCT [11]. The basic physiology of magnesium is depicted in Figure 1.

## 3. Regulation

**Figure 2:**
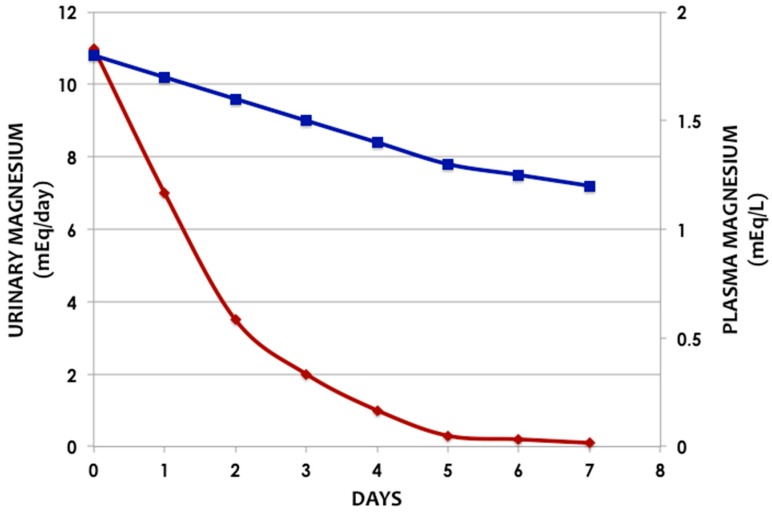
Relation between urinary and plasma magnesium levels in a healthy subject on a magnesium-free diet (Adapted from Shils ME. Experimental human magnesium deficiency [13]) (Blue line—Plasma Magnesium, Red line—Urinary Magnesium).

Magnesium homeostasis is facilitated by intestinal absorption, bone which acts as a reservoir/store, and kidneys which are responsible for magnesium excretion as discussed above. The urinary magnesium excretion increases as there is increased load presented to the kidneys; therefore, sustained hypermagnesemia usually does not occur in the presence of normal renal magnesium excretory function [12]. This relationship is depicted in Figure 2.

## 4. Role at Cellular Level

Magnesium is an essential component of the RNA and DNA tertiary structures. It plays a role in polynucleotide chain binding, and the most studied interaction between magnesium and RNA is transfer RNA (t-RNA) where it is known to stabilize the structure. In DNA, magnesium forms hydrogen bonds to stabilize the DNA conformation [14]. Magnesium is not only required by DNA and RNA polymerases but is also an important factor in DNA repair mechanisms. Magnesium is important in many enzymatic activities. It serves as a cofactor as well as an activator for many enzymes. Some of the enzymes requiring magnesium are topoisomerases, helicases, protein kinases, cyclases, and glycolytic pathway enzymes [14]. One of the important enzymes is adenosine triphosphate, which provides cellular energy for several processes. Magnesium plays a role in the movement of sodium and potassium across membranes [15].

## 5. Causes of Hypomagnesemia

The causes of hypomagnesemia can be broadly classified into three categories: decreased intake, redistribution from extracellular to intracellular, and increased losses via renal or gastrointestinal systems (Table 1).

Decreased intake can result from inadequate dietary consumption, starvation, and alcohol dependence. Approximately 48% of the population in the United States have been shown to consume less than the daily magnesium requirement [16]. Some studies estimate that approximately three-fourths of Americans do not take the recommended dietary allowance of magnesium [1]. The recommended daily allowances for magnesium are outlined in Table 2. Hypomagnesemia develops in people with chronic alcohol abuse who do not satisfy the definition of alcohol dependence.

Refeeding syndrome, hungry bone syndrome, treatment of diabetic ketoacidosis, and acute pancreatitis result in shifts of magnesium from extracellular to intracellular compartments, resulting in hypomagnesemia. The mechanism of hypomagnesemia associated with refeeding syndrome is not clear; it is possibly related to the intracellular movement of magnesium with carbohydrate feeding and preexisting low magnesium status [17]. Hungry bone syndrome causes hypomagnesemia by increased uptake of magnesium by renewing bone after parathyroidectomy or thyroidectomy [18]. Correction of diabetic ketoacidosis also causes hypomagnesemia by driving magnesium into the cells. Acute pancreatitis causes hypomagnesemia by saponification of magnesium in necrotic fat [18].

Gastrointestinal causes of hypomagnesemia include losses due to diarrhea, vomiting, nasogastric suction and fistulas, malabsorption, and small bowel bypass surgery. Proton pump inhibitors (PPIs) are being widely and over-utilized these days; they rank among the top prescribed and one of the most sold drugs. The duration of treatment in most occasions is for months to years. The exact mechanism of PPIs causing hypomagnesemia is unclear but one of the hypotheses is impairment of intestinal absorption [20]. A decrease in intestinal luminal pH with the use of PPIs may alter TRPM6/TRPM7 channel affinity for Mg and disrupt the active transport system. The U.S. Food and Drug Administration (FDA) has received reports of hypomagnesemia with prolonged PPI use [21]. A meta-analysis done by Cheungpasitporn et al. reported the relative risk of hypomagnesemia with PPI use at 1.43 [22]. Primary intestinal hypomagnesemia is a rare disorder and is an inborn error of metabolism. This disease is characterized by a selective defect in magnesium absorption [18]. 

Renal losses are primarily due to defects in the magnesium excretory pathways. They can be secondary to inherited or acquired causes. A few examples of inherited disorders that result in urinary magnesium wasting are Bartter syndrome, Gitelman syndrome, and familial hypomagnesemia with hypercalciuria and nephrocalcinosis (FHHNC). Acquired causes of renal losses include medications and alcohol dependence. Loop and thiazide diuretics inhibit net magnesium reabsorption by inhibition of the electrical gradient required for magnesium reabsorption in the thick ascending loop [2]. On the other hand, the potassium-sparing diuretics lower magnesium excretion by increasing magnesium transport. The nephrotoxic drugs that are known to cause urinary magnesium wasting are aminoglycoside antibiotics, amphotericin B, cisplatin, pentamidine, tacrolimus, and cyclosporine. They cause hypomagnesemia by impairment of loop and distal magnesium reabsorption [18]. Hypomagnesemia is common in alcoholic patients with a prevalence of around 30% and is mostly secondary to excess urinary excretion of magnesium due to tubular dysfunction induced by alcohol [18]. Hypercalcemia can cause hypomagnesemia by increased filtered calcium load to the loop of Henle, resulting in decreased reabsorption of magnesium [18].

## 6. Clinical Presentation/Complications of Hypomagnesemia

The clinical picture of hypomagnesemia can vary from asymptomatic presentation to life-threatening arrhythmias. The important clinical manifestations of hypomagnesemia as outlined in Table 3 include neuromuscular symptoms like muscle weakness, tremors, seizures, and paresthesias; cardiovascular abnormalities like torsade de pointes, ventricular fibrillation, and hypertension; and metabolic abnormalities like hypokalemia and hypocalcemia.

## 7. Neuromuscular/Neurological Manifestations

The earliest manifestations of magnesium deficiency are commonly seen in the form of neuromuscular and neuropsychiatric alterations. The most common clinical manifestations result from hyper-excitability, including positive Chvostek’s and Trousseau’s signs, tremor, fasciculation, and tetany [9]. It can also cause headaches, seizures, fatigue, generalized fatigue, and asthenia.

There may be different mechanisms which can lead to neuromuscular problems in magnesium deficiency. Magnesium is necessary for axon stabilization. The threshold for axon stimulation is decreased and there is an increase in the nerve conduction velocity with hypomagnesemia. It inhibits the entry of calcium into the presynaptic nerve terminals, influencing the release of neurotransmitters at the neuromuscular junction and thereby causing hyper-responsive neuromuscular activity. Magnesium also increases the release of calcium from the sarcoplasmic reticulum and decreases the re-uptake of calcium; these factors lead to increased contractility to a given stimulus and decreased ability to recover from the contraction [9].

## 8. Cardiovascular Manifestations

### 8.1. Atherosclerotic Vascular Disease/Coronary Artery Disease

Magnesium deficiency has been known to be associated with atherosclerotic disease in epidemiological and experimental trials. Its role is suggested in the initiation, morbidity, and mortality associated with infarction of the myocardial cells. Magnesium deficiency causes endothelial dysfunction, a hypercoagulable state, and increased lipid deposition. It can also cause hyper-reactivity of coronary arteries to vasoconstrictive stimuli. It is recommended that magnesium levels be evaluated in patients with vascular disease [23].

Some clinical trials have shown fewer arrhythmias and a mortality reduction after magnesium administration in patients with acute myocardial infarction [24]. In experimental models, it was shown to prevent myocardial stunning and to result in a reduction in ischemic area [25]. On the other hand, patients suffering from uncomplicated acute myocardial infarction or a few hours after the critical point did not benefit from magnesium administration [26].

#### 8.1.1. Arrhythmias

The mechanism underlying the association of arrhythmias and hypomagnesemia is not well understood. The likely physiological mechanism is modulation/regulation of calcium channels and the sodium–potassium membrane pump. A low cytosolic magnesium concentration results in outward movement of potassium, resulting in shortened action potential and increased susceptibility to cardiac arrhythmias [27].

Different electrocardiographic findings with magnesium depletion range from widening of the QRS complex and peaked T-waves with modest depletion, while PR prolongation, progressive QRS widening, and diminution of T-waves are expected with severe magnesium depletion [18]. 

Torsades de pointes is a repetitive polymorphic ventricular tachycardia with QT prolongation associated with hypomagnesemia. Intravenous magnesium is the treatment of choice as per the American Heart Association recommendations.

Magnesium deficiency can cause digoxin toxicity as the sodium potassium ATPase enzyme, which is inhibited by digoxin, is a magnesium-dependent enzyme.

#### 8.1.2. Hypertension

The role of magnesium in hypertension became evident after its introduction in the management of preeclampsia/eclampsia. Vasoconstriction and subsequent hypertension have been observed in patients with low magnesium. Low intracellular magnesium levels lead to increased muscle tone by increasing the intracellular calcium concentration. The administration of magnesium resulted in a decrease in the blood pressure. Clinical and experimental data are conflicting and non-conclusive in regards to the role of oral magnesium in management of hypertension [23]. The DASH (dietary approaches to stop hypertension) eating plan is known to provide foods rich in magnesium [28].

#### 8.1.3. Congestive Heart Failure

The role of hypomagnesemia in congestive heart failure is not clear. Hypomagnesemia is noted in less than 10% of patients with mild to moderate heart failure and is more common in severe congestive heart failure [9].

### 8.2. Endocrine Manifestations

#### 8.2.1. Altered Glucose Homeostasis/Diabetic Complications

Hypomagnesemia is known to be found with increased frequency in patients with type 2 diabetes. It is reported to occur in 13.5–47.7% of patients with type 2 diabetes in comparison to in 2.5–15% of patients without diabetes. Apart from the increased frequency of hypomagnesemia in diabetes patients, it also is known to have an inverse relationship with glycemic control. Magnesium deficiency has been shown to affect glycemic control by way of altered cellular glucose transport, reduced pancreatic insulin secretion, and defective post-receptor insulin signaling. There have also been significant data linking hypomagnesemia to various diabetic complications [29]. The American Diabetes Association published a consensus statement in 1992 suggesting that diabetic patients with hypomagnesemia should receive magnesium supplementation.

Hypomagnesemia has been shown to be an independent predictor for the development of post-transplant diabetes in kidney transplant recipients [30].

#### 8.2.2. Osteoporosis

Magnesium deficiency has been known to be cause osteoporosis. Trabecular bone is lower in magnesium content in people with osteoporosis. The mechanism by which hypomagnesemia leads to osteoporosis is not clear. Different possible theories include low magnesium content altering the structure of apatite crystals, reduction in the levels of parathyroid horme (PTH), end-organ resistance to PTH, and a decrease in vitamin D [31]. 

### 8.3. Magnesium and Asthma

The role of magnesium in asthma is not clear, although many studies have found hypomagnesemia in chronic stable asthmatics as well as during asthma exacerbations [32,33,34]. 

Magnesium exerts its effects on smooth muscle cell contractility, and its deficiency can lead to bronchoconstriction. The bronchodilatory property of magnesium helps in the treatment of acute asthma exacerbations [35].

### 8.4. Nephrolithiasis

Hypomagnesuric calcium nephrolithiasis was found in 6.8% of the 1270 patients that were studied for recurrent nephrolithiasis, suggesting that decreased magnesium excretion may be a contributing factor in the pathogenesis of kidney stones [36].

### 8.5. Magnesium and Pregnancy 

Magnesium is used for the treatment of pre-eclampsia and eclampsia. Pre-eclampsia is a disorder of pregnancy characterized by hypertension and proteinuria. Eclampsia is the occurrence of one or more convulsions associated with pre-eclampsia. Magnesium sulfate is now the drug of choice for women with eclampsia and is better than antiepileptic drugs. The Magpie trial was a randomized controlled trial comparing magnesium sulfate with a placebo for pre-eclampsia. The results demonstrated a reduction by about 50% in the risk of eclampsia in the pre-eclamptic women [37].

### 8.6. Biochemical Manifestations

#### 8.6.1. Hypokalemia

Hypokalemia is a common finding in patients with hypomagnesemia. Potassium depletion in these cases cannot be resolved until magnesium is replete. The mechanism for the hypokalemia in magnesium deficiency can be secondary to multiple mechanisms. One of the mechanisms is magnesium regulating the activity of the ROMK channel (renal outer medullary potassium channel). This channel is the inwardly rectifying potassium channel on the apical surface of the distal nephron which causes a back leak of potassium. A high intracellular magnesium level blocks the ROMK channel pore and prevents potassium efflux. Therefore, low intracellular magnesium will cause potassium efflux and result in hypokalemia [38]. Other possible mechanisms are likely linked to the dependence of sodium-potassium^+^-ATPase, Sodium/Potassium co-transport, and other transport processes on magnesium [39].

#### 8.6.2. Hypocalcemia

Hypocalcemia is also a common manifestation in hypomagnesemia. Symptomatic hypocalcemia is seen more commonly in patients with moderate to severe magnesium deficiency. Hypocalcemia with magnesium deficiency cannot be treated or corrected with calcium, vitamin D, or both. Magnesium therapy alone will resolve the hypocalcemia. Different mechanisms have been hypothesized for hypocalcemia in magnesium deficiency. Some of these factors are impaired secretion of PTH, end-organ resistance for PTH, increase in metabolism of PTH, decrease in 1,25 dihydoxy-vitamin D, etc. [9].

## 9. Diagnostic Aspects

### 9.1. Types of Laboratory Tests

The most commonly used laboratory test for the evaluation of magnesium status is the serum magnesium concentration (SMC). Other clinical laboratory tests are measurement of the serum ionized magnesium concentration and twenty-four-hour urinary magnesium excretion. Research tests include the magnesium retention test, red blood cell magnesium concentration, and tissue magnesium concentration [40].

### 9.2. Serum Magnesium Concentration and Variables

Since only less than 1% of the total body magnesium is present in serum, the SMC does not correlate with and is a poor predictor of the total body or intracellular magnesium content [41]. People within the reference range of the SMC may have a deficit in total body magnesium. The reverse is also possible wherein there is a low SMC with normal magnesium body content [40]. However, in today’s clinical medicine, the SMC is the most feasible and the standard way for quick assessment of magnesium status. 

There is a linear relationship between albumin and SMC at high and low albumin concentrations; however, within the reference interval of albumin, the SMC is independent of the albumin level [40].

Serum magnesium may be higher in vegetarians and vegans in comparison to those with omnivorous diets. It may be lower after endurance exercises or in the third trimester of pregnancy [41].

Serum magnesium may falsely be high if measured after administration of a magnesium dose as magnesium equilibrium between serum and tissues is slow.

### 9.3. Serum Magnesium Concentration Reference Interval

The reference interval for serum magnesium used currently is based on the NHANES I study which studied around 15,000 individuals aged between 18 and 74 years with atomic absorption spectrophotometry. The reference interval was identified as 0.75 mmol/L to 0.955 mmol/L with the mean being 0.85 mmol/L [40].

### 9.4. Urinary Magnesium Excretion

To distinguish between renal and gastrointestinal loss of magnesium, either a 24-h urinary excretion of magnesium or fractional excretion of magnesium with a random urine sample can be performed [18]. A 24-h excretion of more than 10 to 30 mg or a fractional excretion of magnesium above 2% in patients with normal renal function is suggestive of renal magnesium wasting [18]. A low value suggests intake or absorption etiology. The test is cumbersome as it requires a reliable and complete 24-h sample. 

## 10. Treatment

The type of magnesium supplementation, route of administration and aggressiveness depends on the etiology, symptomatology, severity, and other associated electrolyte abnormalities. Patients with simultaneous electrolyte abnormalities such as hypokalemia or hypocalcemia should be treated appropriately with potassium or calcium replacements, respectively, as treatment with magnesium alone will take several days to correct. In any form of magnesium replacement, renal function should be considered and appropriate renal dosing should be done to avoid hypermagnesemia in patients with chronic kidney disease. In all cases, the underlying cause of magnesium deficiency needs to be delineated and appropriately addressed to prevent recurrences [42]. Potassium-sparing diuretics like amiloride increase magnesium reabsorption in the collecting tubule, thereby decreasing magnesium excretion, and can be beneficial especially in cases where diuretic therapy is needed [18].

Patients with hypomagnesemia and on PPIs should consider discontinuing PPIs and switching to alternative medical treatments. Some cases were shown to normalize their magnesium levels after one week of discontinuation of PPIs [21]. Proton Pump Inhibitors were added to the 2015 AGS (American Geriatrics Society) Beers Criteria as potentially inappropriate in older adults [43]. It is suggested that healthcare professionals consider obtaining the serum magnesium level prior to the initiation of PPIs as well as performing periodic checks in patients who are expected to need long-term treatment with these agents. 

Magnesium is present in plant and animal foods. Green leafy vegetables, legumes, nuts, seeds, and whole grains are examples of good sources. Food processing like refining grains can lower the magnesium content [19]. Selected food sources of magnesium with their percent daily values are shown in Table 4.

In severe and symptomatic hypomagnesemia, intravenous magnesium sulfate is the recommended treatment [42], which should be given slowly with clinical and hemodynamic monitoring. An important physiological aspect that must be considered here is that the plasma magnesium concentration is the major regulator of magnesium reabsorption in the loop of Henle, and a rapid elevation in plasma magnesium will result in significant urinary excretion of magnesium (approximately half of the infused magnesium) [18]. This is well illustrated in Figure 2 which shows the relation between urinary and plasma magnesium levels in a healthy subject on a magnesium-free diet [13]. For mild to moderate forms or in asymptomatic patients, oral magnesium is the preferred choice of replacement. Therefore, it is recommended that when intravenous magnesium replacement is needed, the rate of infusion should be slow over 12–24 h.

There are very limited human data on the bioavailability of different magnesium supplementation salts. A study done in rats showed that organic salts are more bioavailable than inorganic ones, with magnesium gluconate having the highest bioavailability. Magnesium gluconate exhibited the highest Mg bioavailability of the ten Mg salts studied [45]. There are different oral formulations with varying bioavailabilities, magnesium content, and tolerance. Table 5 shows the different magnesium salts, their magnesium contents, bioavailabilities, and tolerabilities. 

## Figures and Tables

**Table 1:** Causes of hypomagnesemia.

**Decreased Intake**
Decreased Dietary consumption
Alcohol Dependence
Parenteral Nutrition
**Redistribution from Extracellular to Intracellular Compartment:**
Refeeding Syndrome
Hungry Bone Syndrome
Treatment of Diabetic Ketoacidosis
Acute Pancreatitis
**Gastrointestinal Losses:**
Diarrhea
Vomiting
Nasogastric suction
Fistulas
Malabsorption
Small bowel bypass surgery
Proton Pump Inhibitors
**Renal Losses:**
***Familial:***
Bartter syndrome, Gitelman syndrome, Familial hypomagnesemia with hypercalciuria and nephrocalcinosis (FHHNC)
***Acquired:***
Medications: Thiazide Diuretic, Aminoglycoside Antibiotics, Amphotericin B, Cisplatin, Pentamidine, Tacrolimus, Cyclosporine
Alcohol Dependence, Hypercalcemia

**Table 2:** Recommended dietary allowances (RDAs) for magnesium in mg/day [19].

Age	Male	Female	Pregnancy	Lactation
19–30 years	400	310	350	310
31–50 years	420	320	360	320
>51 years	420	320		

**Table 3:** Clinical manifestations of hypomagnesemia.

**Neuromuscular/Nervous System:**
Positive Chvostek’s And Trousseau’s Signs, Tremor, Fasciculations, Tetany, Headaches, Seizures, Fatigue, Generalized Fatigue, Asthenia
**Cardiovascular:**
***Atherosclerotic Vascular Disease/Coronary Artery Disease***
***Arrhythmias:*** Torsades de pointes, PR prolongation, progressive QRS widening and diminution of T-waves
***Hypertension***
***Congestive Heart Failure***
**Endocrine: *Altered Glucose Homeostasis/Diabetic Complications***
***Osteoporosis***
**Biochemical/Others:**
Hypokalemia
Hypocalcemia
Asthma
Nephrolithiasis

**Table 4:** Selected food sources of magnesium [44].

Food	Milligrams per Serving	Percent Daily Value
Almonds, dry roasted, 1 ounce	80	20
Spinach, boiled, ½ cup	78	20
Cashews, dry roasted, 1 ounce	74	19
Peanuts, oil roasted, ¼ cup	63	16
Cereal, shredded wheat, 2 large biscuits	61	15
Soymilk, plain or vanilla, 1 cup	61	15
Black beans, cooked, ½ cup	60	15

**Table 5:** Different magnesium formulations.

Magnesium Supplement	Elemental Magnesium (Percent)	Bioavailability (as Fractional Absorption of the Administered Dose)	Bioavailability (Relative Comparison)	Tolerability (Diarrhea)
Magnesium Oxide	60	4%	Extremely low	++
Magnesium Carbonate	45	*	Extremely low	*
Magnesium Hydroxide	42	4%	*	++
Magnesium Citrate	16	12%	Good	++
Magnesium Lactate	12	12%	Excellent	+
Magnesium Chloride	12	12%	Good	+
Magnesium Aspartate	10	*	*	*
Magnesium Sulfate	10	4%	*	++
Magnesium Gluconate	5	*	Good	±

Data obtained from Guerrera et al [46], Firoz M [47], Ranade [48], Epocrates [49], The Schrier Atlas of Diseases of the Kidney [50]. * Data could not be obtained. ++ Indicates higher incidence of diarrhea, + indicates lesser incidence of diarrhea. ± Indicates equivocal incidence of diarrhea.
